# The enigma of Bálint's syndrome: neural substrates and cognitive deficits

**DOI:** 10.3389/fnhum.2014.00123

**Published:** 2014-03-07

**Authors:** Magdalena Chechlacz, Glyn W. Humphreys

**Affiliations:** Department of Experimental Psychology, University of OxfordOxford, UK

**Keywords:** Bálint's syndrome, simultanagnosia, optic ataxia, ocular apraxia, posterior cortical atrophy

In 1909 a Hungarian physician, Rezsö Bálint, published the first report of a striking visual problem in a patient with bilateral parietal lesions (Bálint, 1909). Bálint had studied his remarkable patient for several years, starting in 1903, struggling to fully understand the nature of the complex neurological disorder. The disorder was characterized by problems in simultaneously processing multiple items resulting in poor interpretation of complex visual scenes, sensory inattention, and deficits in visually guided limb and eye movements (Bálint, 1909). While Balint's subsequent clinical work took him away from neurology to general medicine, the 1909 case study remains his lasting legacy and helped to define a syndrome now named after him (Husain and Stein, 1988; Rizzo and Vecera, 2002). Since Balint's first publication, several other cases have been published and in its current status the syndrome is described to comprise of several primary symptoms including simultanagnosia (impaired spatial awareness of more than one object at time), optic ataxia (misreaching to visual targets), ocular apraxia (described by Bálint as “psychic paralysis of gaze”) and general visuospatial disorientation (Bálint, 1909; Wolpert, 1924; Rizzo and Vecera, 2002). Affected individuals often behave as if they are blind due to the lack of normal visual awareness of the surrounding world, which for them can appear to be a chaotic compilation of random single objects. The disorder has a severe impact on everyday living. Simple and previously easy tasks such as walking or eating and drinking become very difficult due to the patient perceiving only one object at a time, failing to appreciate the relationships between different items and having problems with reaching and grasping objects. Not surprisingly given what the disorder might tell us about spatial awareness, object perception and action, Bálint's syndrome has captivated the interest of researchers aiming to understand the underlying functional and neural processes.

There are many interesting questions to raise about the syndrome. First, the individual symptoms of the syndrome not only dissociate but also represent very wide categories of deficits often difficult to assess with standard neuropsychological tests and varying significantly between individual cases (Damasio et al., 2000; Rizzo and Vecera, 2002; Jackson et al., 2005). Secondly, while the original case reported by Bálint occurred following bilateral parietal lesions, different lesion combinations have been described in the literature (for review see Rizzo and Vecera, 2002; Chechlacz et al., 2012). Finally, the syndrome has been reported to result from diverse aetiologies including bilateral strokes, intracranial tumors, traumatic brain damage and neurodegenerative conditions including posterior cortical atrophy (PCA) and Alzheimer's disease (Benson et al., 1988; Hof et al., 1990; Damasio et al., 2000; Rizzo and Vecera, 2002). The syndrome is infrequent and each individual case offers unique perspectives into the cognitive and neural mechanisms underlying the disorder. The aim of this special issue then is to bring together into a single forum current research on the syndrome. The studies collected here cover both the anatomical and the cognitive mechanisms of the different symptoms associated with the syndrome.

The most striking and most widely studied symptom associated with Bálint's syndrome is simultanagnosia. Simultanagnosia was defined by Wopert as an inability to interpret complex visual displays due to problems with processing multiple items and the relations between them (Wolpert, 1924). Dalrymple et al. (2013) provide here a review of processing deficits in simultanagnosia focusing on object-based versus space-based attention. Two further articles in this Special Issue (Meek et al., 2013a; Shakespeare et al., 2013) examine deficits in complex scene perception in PCA—a disorder in which the clinical presentation often encompasses Bálint's syndrome as its key feature.

Simultanagnosia has been frequently associated with deficits in global processing when the integration of multiple local elements into global compound shapes is required (e.g., Karnath et al., 2000; Shalev et al., 2005). Interestingly, though the patients are typically poor at explicitly reporting global compound shapes, there is evidence that global processing may take place implicitly. Here, Mevorach et al. (2013) report a case study of a patient who was able to respond accurately to both global and local targets when they were presented as the salient aspects of the compound letter, but the patient was then unable to report the alternative level. The complex nature of global/local processing and the potential mechanisms contributing to deficits observed in simultanagnosic patients are also addressed here by two other studies, which examine the cognitive and neural substrates of Gestalt perception in healthy individuals (Rennig et al., 2013a,b). The first study by Rennig et al. (2013b) demonstrates the important role of size constancy for hierarchical object processing (the integration of local elements into global objects) using a paradigm based on the Kanizsa illusion. The second paper (Rennig et al., 2013a) compares BOLD responses in chess experts vs chess novices using ROI analyses of data from four fMRI studies. This paper elegantly demonstrates that the temporo-parietal junction (a region frequently reported to be damaged in simultanagnosic patients) plays a critical role in the processing of complex, learned visual stimulus configurations (Rennig et al., 2013a).

Optic ataxia, defined as an impairment in visually guided reaching, is one of the constituent symptoms of Bálint's syndrome following bilateral parietal lesions but the disorder is also often reported in isolation of Bálint's syndrome in patients with unilateral lesions to the superior parietal lobule of either hemisphere (e.g., Auerbach and Alexander, 1981; Ferro, 1984; Perenin and Vighetto, 1988; Karnath and Perenin, 2005). Cavina-Pratesi et al. (2013) here present a thorough examination of deficits in visually guided action in a patient with unilateral extensive lesion within left posterior parietal cortex (PPC). Based on their single case analysis these authors draw a model of the functional organization of the PPC (subdivided based on specific end-goals such as reaching, grasping, or looking). The second case study included here is by Khan et al. (2013), who examined optic ataxia in the presence of blind field (hemianopia) using a saccadic visual updating paradigm. This paradigm aims to examine whether and how information viewed in the intact visual field is remapped into the blind field and then used for object reaching. Similarly to simultanagnosia, optic ataxia has been reported in PCA patients and Meek et al. (2013b) provide a comprehensive evaluation of reaching and grasping deficits in this clinical population. Importantly, the authors contrast deficits associated with optic ataxia resulting from PCA and visuomotor problems reported in other clinical populations with optic ataxia. Difficulties in understanding the symptoms of Bálint's syndrome, including optic ataxia, also arise from the fact that many studies are based on single cases and the paucity of standard neuropsychological testing tools result in problems with generalizing case findings. In view of that, Borchers et al. (2013) discuss the guidelines for the clinical diagnosis of optic ataxia with the aim being to move one step closer to developing standardized diagnostic tools and thus improving comparisons between different studies.

Poor initiation of saccadic eye movements is characteristic of ocular apraxia, the third and the least documented of the components of Bálint's syndrome. Bálint described this symptom as an inability to shift gaze voluntarily despite intact eye rotation (Bálint, 1909). Based on his original report as well as on later described cases, it is not entirely clear whether the “psychic paralysis of gaze” as originally described by Bálint occurs on its own without other visuospatial deficits and depends solely on parietal damage (Rizzo and Vecera, 2002). Ptak and Muri (2013) here provide a comprehensive review of the role of the parietal cortex in saccade planning, contrasting (i) the findings from behavioral analyses combined with lesion mapping in patients with unilateral parietal lesions (spatial neglect patients) and (ii) impairments in saccade planning in patients with bilateral parietal lesions (Bálint's patients).

The papers compiled in this special topic dedicated to Bálint's syndrome show that, despite the hundred years since Rezsö Bálint was first puzzled by the complex and severe visuospatial deficits in his patient, the analysis of the syndrome continues and we are still some way from reaching definitive conclusions. The current papers highlight each of the symptoms that characterize the syndrome and thus, even in an indirect way, question the extent to which the syndrome is unitary (see also Rizzo, 1993; Damasio et al., 2000; Rizzo and Vecera, 2002). A major question for future research is whether, when there are co-occurring deficits, they are functionally related or not. The overlap and dissociations between the symptoms of this complex syndrome will remain an important topic for research for some time to come.
